# Laparoscopic Left Liver Sectoriectomy of Caroli's Disease Limited to Segment II and III

**DOI:** 10.3791/1118

**Published:** 2009-02-27

**Authors:** Luigi Boni, Gianlorenzo Dionigi, Francesca Rovera, Matteo Di Giuseppe

**Affiliations:** Minimally Invasive Surgery Resarch Center, Department of Surgical Sciences, University of Insubria; Endocrine Surgery Research Center, University of Insubria

## Abstract

Caroli's disease is defined as a abnormal dilatation of the intra-hepatica bile ducts: Its  incidence is extremely low  (1 in 1,000,000 population) and in most of the cases the whole liver is interested and liver transplantation is the treatment of choice.  In case of dilatation limited to the left or right lobe, liver resection can be performed. For many year the standard approach for liver resection has been a formal laparotomy by means of a large incision of abdomen that is characterized  by significant post-operatie morbidity. More recently, minimally invasive, laparoscopic approach has been proposed as possible surgical technique for liver resection both for benign and malignant diseases. The main benefits of the minimally invasive approach is represented by a significant reduction of the surgical trauma that allows a faster recovery a less post-operative complications.

This video shows a case of Caroli s disease occured in a 58 years old male admitted at the gastroenterology department for sudden onset of abdominal pain associated with fever (>38C° ), nausea and shivering.  Abdominal ultrasound demonstrated a significant dilatation of intra-hepatic left sited bile ducts with no evidences of gallbladder or common bile duct  stones. Such findings were confirmed abdominal high resolution computer tomography. Laparoscopic left sectoriectomy was planned. Five trocars and 30° optic was used, exploration of the  abdominal cavity showed no adhesions or evidences of other diseases.

In order to control blood inflow to the liver, vascular clamp was placed on the hepatic pedicle (Pringle s manouvre), Parenchymal division is carried out with a combined use of 5 mm bipolar forceps and 5 mm ultrasonic dissector. A severely dilated left hepatic duct was isolated and divided using a 45mm endoscopic vascular stapler. Liver dissection was continued up to isolation of the main left portal branch that was then divided with a further cartridge of 45 mm vascular stapler.

At his point the left liver remains attached only by the left hepatic vein: division of the triangular ligament was performed using monopolar  hook and the hepatic vein isolated and the divided using vascular stapler.

Haemostatis was refined by application of argon beam coagulation and no bleeding was revealed even after removal of the vascular clamp (total Pringle s time 27 minutes).

Postoperative course was uneventful, minimal elevation of the liver function tests was recorded in post-operative day 1 but returned to normal at discharged on post-operative day 3.

**Figure:**
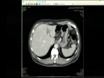


## Protocol

### Preoperative Workout

1. In the abdominal CT scan it can be easily seen the bile ducts (appearing inside the liver parenchyma with a darker color) widely enlarged on the left side of the liver .3D reconstruction demonstrated the presence of a right hepatic artery originating directly from the aorta and no further vascular abnormalities.
2. The liver can be functionally divided into 8 segments as shown in the video and considering that the dilatation was limited to segment 2 and 3 (left sectors), and a laparoscopic left sectoriectomy was planned.

### Intraoperative procedure

1. The patient is placed in supine position and the surgeon stand between patient’s legs. 5 trocars are used and placed as shown in the video
2. Vascular clamp is placed on the portal triad in order to reduce bleeding (Pringle’s manouvre)
3. Division of the liver parenchyma is performed using a combination or bipolar electrocautery and harmonic scalpel along the falciform ligament between segment III and IV
4. Once the segment III pedicle is fully dissected it can be divided using a 45mm endo GIA with a vascular cartridge.
5. he procedure carries on with further division of the parenchyma till the main pedicle to segment II is reached, isolated and finally divided using a 45mm endoGIA with vascular cartridge
6. The left triangular ligament is now divided using electrocautery and the left hepatic vein is isolated and divided using vascular stapler
7. The liver surface is checked for bleeding; argon beam is applied on the liver surface and the vascular clamp is removed. At this point fibrin glue is applied on order to reduce the risk of post-operative bile leaks. The specimen is placed in endobag and removed

## Discussion

Laparoscopic surgery can be safely performed also for liver resection both for benign and malignant diseases. Reducing surgical trauma using minimally invasive approach allows to minimize the incidence of post-operative complications and post-operative hospital stay.
